# Next release of the European Marine Omics Biodiversity Observation Network (EMO BON) shotgun metagenomic data from water and sediment samples (Release 2)

**DOI:** 10.3897/BDJ.14.e178484

**Published:** 2026-01-22

**Authors:** Ioulia Santi, Christina Pavloudi, Maria Abagnale, Iñigo Azua, Zuriñe Baña, Mauro Bastianini, Caroline Belser, Kristel Berg, Jone Bilbao, Kimberley Bird, Caroline Broudin, Mathieu Camusat, Ibon Cancio, Louis Caray-Counil, Raffaella Casotti, Jade Castel, Thierry Comtet, Cymon J Cox, Michael Cunliffe, Claire Daguin, Klaas Deneudt, Oihane Díaz de Cerio, Katrina Exter, Cécile Fauvelot, Yann Fontana, Miquel J Frada, Pierre E Galand, Roberto Gallia, Laurence Garczarek, Jose González Fernández, Laure Guillou, Hanneloor Heynderickx, Gil Koplovitz, Celine Labrune, Rune Lagaisse, Arnaud Laroquette, Lyvia Lescure, Eva Lopes, Melina Loulakaki, Bruno Louro, Catarina Magalhães, Francesca Margiotta, Hannah Moal, Alice Moussy, Fabrice Not, Isabella Percopo, Estefanía Paredes Rosendo, Erwan Péru, Julie Poulain, Kim Praebel, Fabienne Rigaut-Jalabert, Sarah Romac, Jadwiga Rzeznik-Orignac, Diana Sarno, Jesús Souza Troncoso, Eric Thiébaut, Wilfried Thomas, Andrzej Tkacz, Ferdinando Tramontano, Anna Chiara Trano, Patrick Wincker, Nicolas Pade

**Affiliations:** 1 European Marine Biological Resource Centre (EMBRC-ERIC), Paris, France European Marine Biological Resource Centre (EMBRC-ERIC) Paris France; 2 Hellenic Center for Marine Research (HCMR), Institute of Marine Biology Biotechnology and Aquaculture (IMBBC), Heraklion, Greece Hellenic Center for Marine Research (HCMR), Institute of Marine Biology Biotechnology and Aquaculture (IMBBC) Heraklion Greece; 3 Stazione Zoologica Anton Dohrn (SZN), Naples, Italy Stazione Zoologica Anton Dohrn (SZN) Naples Italy; 4 Department of Immunology, Microbiology & Parasitology, University of the Basque Country (UPV/EHU), Plentzia, Spain Department of Immunology, Microbiology & Parasitology, University of the Basque Country (UPV/EHU) Plentzia Spain; 5 Plentzia Marine Station (PiE-UPV/EHU), University of the Basque Country (UPV/EHU), Plentzia, Spain Plentzia Marine Station (PiE-UPV/EHU), University of the Basque Country (UPV/EHU) Plentzia Spain; 6 Institute of Marine Sciences (ISMAR) National Research Council of Italy (CNR), Venice, Italy Institute of Marine Sciences (ISMAR) National Research Council of Italy (CNR) Venice Italy; 7 Génomique Métabolique, Genoscope, Institut François Jacob, CEA, CNRS, Univ Evry, Université Paris-Saclay, Evry, France Génomique Métabolique, Genoscope, Institut François Jacob, CEA, CNRS, Univ Evry, Université Paris-Saclay Evry France; 8 Norwegian College of Fishery Science, UiT The Arctic University of Norway, Tromsø, Norway Norwegian College of Fishery Science, UiT The Arctic University of Norway Tromsø Norway; 9 Department of Plant Biology and Ecology, University of the Basque Country (UPV/EHU), Plentzia, Spain Department of Plant Biology and Ecology, University of the Basque Country (UPV/EHU) Plentzia Spain; 10 The Marine Biological Association, Plymouth, United Kingdom The Marine Biological Association Plymouth United Kingdom; 11 Sorbonne Université, CNRS, FR2424, Station Biologique de Roscoff, 29680 Roscoff, France Sorbonne Université, CNRS, FR2424, Station Biologique de Roscoff 29680 Roscoff France; 12 CBET+ Research Group, Department of Zoology & Animal Cell Biology, University of the Basque Country (UPV/EHU), Biscay, Spain CBET+ Research Group, Department of Zoology & Animal Cell Biology, University of the Basque Country (UPV/EHU) Biscay Spain; 13 Sorbonne Université, CNRS, Laboratoire Océanographique de Villefranche-sur-mer, Villefranche sur mer, France Sorbonne Université, CNRS, Laboratoire Océanographique de Villefranche-sur-mer Villefranche sur mer France; 14 Sorbonne Université, CNRS, UMR7144 AD2M, Station Biologique de Roscoff, 29680 Roscoff, France Sorbonne Université, CNRS, UMR7144 AD2M, Station Biologique de Roscoff 29680 Roscoff France; 15 Centro de Ciências do Mar do Algarve (CCMAR/CIMAR LA), Universidade do Algarve, Faro, Portugal Centro de Ciências do Mar do Algarve (CCMAR/CIMAR LA), Universidade do Algarve Faro Portugal; 16 School of Biological and Marine Sciences, University of Plymouth, Plymouth, United Kingdom School of Biological and Marine Sciences, University of Plymouth Plymouth United Kingdom; 17 Flanders Marine Institute (VLIZ), Ostend, Belgium Flanders Marine Institute (VLIZ) Ostend Belgium; 18 Interuniversity Institute for Marine Sciences in Eilat, Eilat, Israel Interuniversity Institute for Marine Sciences in Eilat Eilat Israel; 19 Sorbonne Université, CNRS, Laboratoire d'Ecogéochimie des Environnements Benthiques (LECOB), Banyuls sur Mer, France Sorbonne Université, CNRS, Laboratoire d'Ecogéochimie des Environnements Benthiques (LECOB) Banyuls sur Mer France; 20 Centro de Investigación Mariña (CIM), Universidade de Vigo, Vigo, Spain Centro de Investigación Mariña (CIM), Universidade de Vigo Vigo Spain; 21 Faculty of Sciences of University of Porto, Porto, Portugal Faculty of Sciences of University of Porto Porto Portugal; 22 Interdisciplinary Centre of Marine and Environmental Research (CIIMAR), Matosinhos, Portugal Interdisciplinary Centre of Marine and Environmental Research (CIIMAR) Matosinhos Portugal; 23 Genoscope, Institut François Jacob, Commissariat à l'Energie Atomique (CEA), Université Paris-Saclay, Evry, France Genoscope, Institut François Jacob, Commissariat à l'Energie Atomique (CEA), Université Paris-Saclay Evry France; 24 Research Federation for the study of Global Ocean Systems Ecology and Evolution, FR2022/ Tara Oceans-GOSEE, 3 rue Michel-Ange, Paris, France Research Federation for the study of Global Ocean Systems Ecology and Evolution, FR2022/ Tara Oceans-GOSEE, 3 rue Michel-Ange Paris France; 25 Sorbonne Université, CNRS, OSU STAMAR, Station Biologique de Roscoff, 29680 Roscoff, France Sorbonne Université, CNRS, OSU STAMAR, Station Biologique de Roscoff 29680 Roscoff France

**Keywords:** metagenomics, marine biodiversity, ocean observation, water column, soft substrate, microorganisms

## Abstract

The European Marine Omics Biodiversity Observation Network (EMO BON) is a long-term genomic observatory run by the European Research Infrastructure European Marine Biological Resource Centre (EMBRC). It was established in 2021 to support the challenges of biodiversity observation and unsystematic management of biodiversity data in the European seas. EMO BON introduced and coordinated the systematic and harmonised observation of biodiversity amongst more than fourteen marine stations in the European coastline. Here, we report the next release (Release 2) of shotgun metagenomic data from seawater and sediment microbial communities.

## Introduction

The advancement of marine biological observation needs to be built on long-term initiatives, able to produce data tackling not only the spatial, but also the temporal component of the dynamic biodiversity. The coordination across different entities and countries has always presented a challenge and a prerequisite for expanded observation capacity in space and time. Research Infrastructures (RIs), as part of the pan-European scientific landscape, can play a substantial role in this by adding value for their members in providing services and resources, such as management and coordination of marine biological observation.

The EMBRC RI consolidated the marine genomics experience in its community and, therefore, established and coordinated the EMO BON network (Santi et al. 2023). The network widens the distribution of the observatories along the entire European coastline and includes a unique and broad coverage of habitats and longitudes, from the Arctic to the Red Sea. The different observatory stations are coordinated at the RI-level and act as nodes of one integrated network. In 2025, EMO BON is running its fifth year in operation, all the while sample collection and processing, data generation and management and network coordination progress continuously. The aspiration is to maintain the network for at least 10 years of systematic biodiversity observation.

### Value of the dataset

This work is the second set of genomic datasets of the EMO BON long-term observation network. It follows up from the first dataset that was realised in this journal in March 2025 (Pavloudi et al. 2025) and will be followed by future releases. All data releases are accompanied by a data publication describing the (meta)data and their online location and allowing for maximum data re-usability and openness, exhibiting the value and potential impact in biodiversity observation research. This release includes raw shotgun metagenomic sequencing data from the seawater and the marine sediment collected between October and December 2021 from 13 observatories, across the European coast and in the Red Sea. The raw sequence data are deposited in and accessible through the European Nucleotide Archive (ENA) (O’Cathail et al. 2024), together with metadata associated with the sampling event, sample preparation, processing and sequencing procedures and a diverse set of measured environmental variables available, such as temperature, salinity and nutrient concentrations in the associated BioSamples (Courtot et al. 2021). Follow-up publications related to the EMO BON macro- and meio-benthos metabarcoding data are upcoming. In addition, it connects with the recent data release from the marine genetic monitoring programme ARMS-MBON (Autonomous Reef Monitoring Structure Marine Biodiversity Observation Network) (Daraghmeh et al. 2025) that was merged into EMO BON and the ARMS data releases that are in preparation (Pagnier et al. 2025).

The present dataset is part of a larger biodiversity time series dataset that is progressively being released in ENA and published in this journal. The impact of the whole dataset is predicted to appear after at least 5 years of data releases, as the value increases with the extension of the data's temporal range. The existence of a marine biodiversity dataset of this depth and quality, spanning at least 5 years and multiple coastal areas in Europe is expected to have a broad appeal, hence increasing the data users and applications.

In addition, this dataset aligns with the mission of the Ocean Biodiversity Information System (OBIS), to facilitate free and open access to biodiversity data and information on marine life. Data products generated after the bioinformatics analyses of this dataset will include taxonomical and functional information and will be submitted in OBIS and in the Global Biodiversity Information Facility (GBIF), using the DNA extension of the Darwin Core standards. Following the data availability in OBIS and GBIF, the data products will be incorporated in the European Marine Observation and Data Network (EMODnet) and the European Digital Twin of the Ocean (EU DTO). This FAIR data flow into the EU DTO will allow, on one hand, to fill gaps in the current knowledge on biodiversity of the world's oceans and, on the other hand, to model and simulate the ‘what if’ scenarios, further advancing ocean knowledge and providing information for evidence-based policy-making.

The potential arising from the incorporation of genomic methodologies into marine observation is enormous. Out of many arguments outlining the added value of including genomics to the study of biodiversity in scientific publications (for example, Cordier et al. (2020), Pawlowski et al. (2022), Theissinger et al. (2023)), the most relevant and influential to the EMO BON initiative are the ability to early detect non-indigenous species, the identification of cryptic species and early life stages (e.g. larvae), the interaction amongst species, the study of evolutionary ecology and events shaping species diversity, the access to communities difficult to identify morphologically such as microorganisms and meiofauna, the exploration and explanation of biodiversity patterns across time and space including different communities, from microorganisms to macrobenthos and the response of biodiversity to ecosystem changes (for example, climate change and pollution). Furthermore, the metagenomic analyses of samples allow us to access data and, subsequently, information on function. This leads to additional scientific directions like bioprospecting and biotechnology research, community functioning across time and space and connection of communities functioning to ecosystem changes.

In this dataset, each material sample is represented by identical technical replicates, two of which are processed and sequenced using the exact same methodologies and workflow. The duplicate data can increase the quality of the whole sequencing dataset by allowing the assessment of the processing and sequencing procedures and providing an additional level of quality assurance.

The field of DNA-based environmental research is rapidly evolving and, thus, anticipating the future need for biodiversity re-assessments, another two technical replicates for each material sample are stored in the long-term. The long-term stored replicates can be processed in the future using new technologies or targeted scientific questions. Using the long-term stored samples will open a window in time and allow potential re-sequencing or usage of other omics technologies and combining with the data produced in the present.

## Methods

The sampling, sample and data processing methodologies are described in the first EMO BON dataset publication (Pavloudi et al. 2025). The procedures are also briefly described in the following subsections.

### Sampling

Seawater sampling was performed by following the Standard Operating Procedures (SOPs) included in the EMO BON Handbook (Santi et al. 2021) and in particular “WaSOP 1 (basic)”. Subsurface seawater was collected using Niskin bottles, pre-filtered using 200 μm mesh to exclude particles > 200 μm. The pre-filtered seawater was sequentially filtered through 3 μm and 0.2 μm polycarbonate filter membranes; this produced two seawater samples including particles of different size fractions: 3-200 μm and 0.2-3 μm. Each of the membranes was subsequently cut into two equal pieces using a sterile scalpel; each cut membrane was considered to represent one technical replicate. The sequential filtration took place two separate times producing 2 X the 3-200 μm and 0.2-3 μm membranes, which were then cut into two pieces of equal size, finally generating four technical replicates [First sequential filtration: 3-200 μm membrane cut in half to generate 3-200 μm replicates (1) and (2); and 0.2-3 μm membrane cut in half to generate 0.2-3 μm replicates (1) and (2). Second sequential filtration: 3-200 μm membrane cut in half to generate 3-200 μm replicates (3) and (4); and 0.2-3 μm membrane cut in half to generate 0.2-3 μm replicates (3) and (4)]. Replicates (1) and (2) were preserved in individual tubes using the DNA/RNA shield preservative (Zymo Research) and stored at -80^o^C until shipment to the sequencing facility. Replicates (3) and (4) were preserved in cryotubes without the addition of DNA/RNA Shield and stored at -80^o^C for long-term storage.

Sediment was collected by following the Standard Operating Procedures (SOPs) included in the EMO BON Handbook (Santi et al. 2021). In particular, “SoSOP 1 (intertidal sediments)" was followed by the observatories NRMCB and RFormosa, “SoSOP 2 (coastal sediments by diving)" was followed by the observatories ROSKOGO and OOB, “SoSOP 3 (coastal sediments by research vessel)" was followed by the observatory BPNS. In all SOPs, sediment was sampled (or subsampled from the sediment grab in SoSOP 3) using sediment corers. The top 5 cm were sliced, gently homogenised and subsamples were placed in individual tubes to represent four technical replicates. Similarly to the seawater approach, replicates (1) and (2) were preserved in DNA/RNA Shield and stored at -80^o^C until shipment to the sequencing facility; replicates (3) and (4) were preserved in cryotubes without the addition of DNA/RNA Shield and stored at -80^o^C for long-term storage.

#### Geographic range

**Figure 1.**
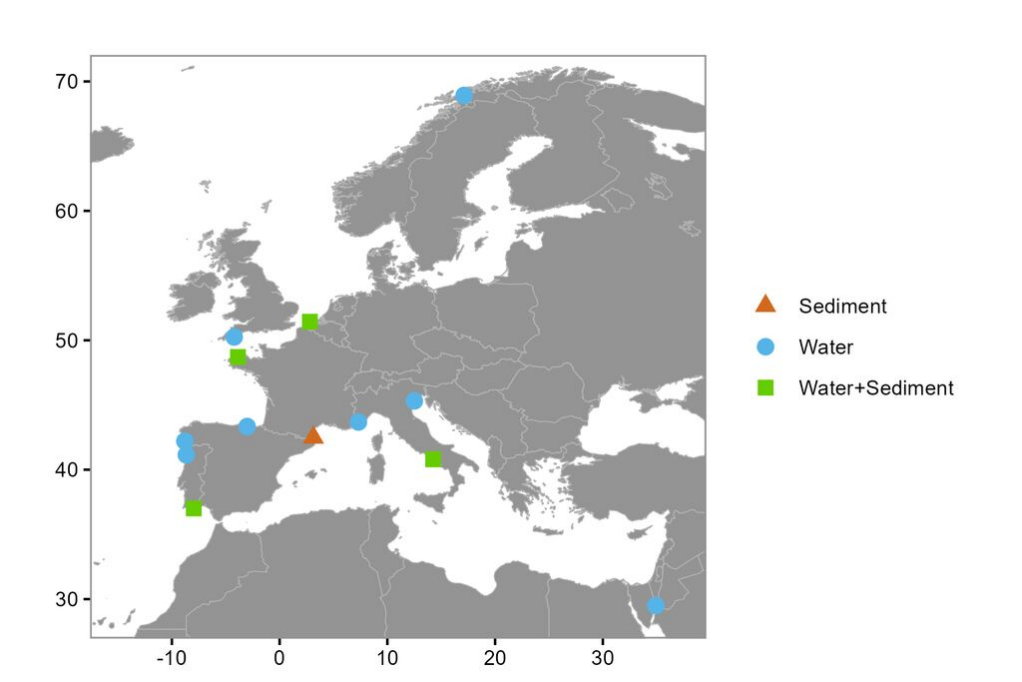
Map of EMO BON observatory sampling sites for sediment (triangle ▲), seawater (circle ●) and both seawater and sediment (square ◼), included in this dataset. Observatory IUIEilat located in the Gulf of Eilat, Indian Ocean, falls outside the primary European focus of this study.

EMO BON observatories are operating in each of the EMBRC member countries in the EU. This dataset includes data from Norway, Belgium, UK (former EMBRC member), France, Spain, Portugal, Italy and Israel. The geographic range of the dataset includes 14 locations across eight marine ecoregions, based on the Marine Ecoregions of the World (MEOW) (Spalding et al. 2007) (Table 1, Fig. 1). The locality of the observatories is also described using standardised georeferenced terms from the Marine Regions (Flanders Marine Institute 2025), from the broader (ocean/sea) to the regional and the local level (Table 1, Fig. 1). This dataset includes data from the observatory IUIEilat located in the Gulf of Eilat, Indian Ocean, which falls outside the primary European focus of this study.

#### Temporal range

This dataset includes samples collected from October to December 2021.

### Sample processing

#### DNA extraction, library preparation and sequencing

DNA extraction, library preparation and sequencing were collectively performed at the Genoscope French National Sequencing Centre and are described in detail in the first EMO BON dataset publication (Pavloudi et al. 2025). Brief description of sample processing are included here. The water column samples were extracted according to Alberti et al. (2017): manual cell disruption by cryogenic grinding of membrane filters followed by chemical lysis and nucleic acid purification using NucleoSpin RNA Kits, combined with the NucleoSpin RNA/DNA buffer set (Macherey-Nagel, Düren, Germany). The sediment samples were extracted using the DNeasy PowerSoil Pro Kit (Qiagen) with slight modifications.

Fragments of ~ 350 bp were obtained by sonication. After freebarcodes adapters were added, ligation products were purified using beads and Illumina specific adapters were added by PCR amplifications (2 PCR reactions, 14 cycles). Libraries were quantified and their size profiles analysed prior to sequencing.

Sequencing was performed using 151-bp pairwise read chemistry on an Illumina NovaSeq6000 sequencer, using S4 Flowcells (Illumina, San Diego, CA, USA). A minimum of 40,000 million useful paired-end reads were obtained per sample. Short Illumina reads were bioinformatically post-processed *sensu* Alberti et al. (2017) to filter out low-quality data. Finally, read pairs mapping to the phage phiX genome were identified and discarded using SOAP aligner (Li et al. (2008), default parameters) and the Enterobacteria phage PhiX174 reference sequence (GenBank: NC_001422.1)

## Biodiversity scope

### Target

Microbial Prokaryotic and Eukaryotic biodiversity in the marine environment.

### Taxonomic range

Archaea, Bacteria, Eukaryota

## Data Resources

Details for the samples can be found in Suppl. material 1. All the raw sequence files of this study were submitted to ENA (O’Cathail et al. 2024) with the umbrella study accession number PRJEB51688. The accession numbers of the component projects under the umbrella study are PRJEB51656, PRJEB51652, PRJEB51653, PRJEB51657, PRJEB50566, PRJEB51664, PRJEB51662, PRJEB51654, PRJEB51660, PRJEB51661, PRJEB51658, PRJEB51665 and PRJEB51659. All sampling events and environmental data, linked to the respective accession numbers, are also available to browse and download from EMO BON’s data landing page.

### Resource 1

Download URL: ftp.sra.ebi.ac.uk/vol1/fastq/ERR148/078/ERR14888678/ERR14888678_1.fastq.gz

Resource identifier: ERR14888678

Data format : FASTQ

Download URL: ftp.sra.ebi.ac.uk/vol1/fastq/ERR148/078/ERR14888678/ERR14888678_2.fastq.gz

### Resource 2

Download URL: ftp.sra.ebi.ac.uk/vol1/fastq/ERR148/080/ERR14888680/ERR14888680_1.fastq.gz

Resource identifier: ERR14888680

Data format : FASTQ

Download URL: ftp.sra.ebi.ac.uk/vol1/fastq/ERR148/080/ERR14888680/ERR14888680_2.fastq.gz

### Resource 3

Download URL: ftp.sra.ebi.ac.uk/vol1/fastq/ERR148/081/ERR14888681/ERR14888681_1.fastq.gz

Resource identifier: ERR14888681

Data format : FASTQ

Download URL: ftp.sra.ebi.ac.uk/vol1/fastq/ERR148/081/ERR14888681/ERR14888681_2.fastq.gz

### Resource 4

Download URL: ftp.sra.ebi.ac.uk/vol1/fastq/ERR148/082/ERR14888682/ERR14888682_1.fastq.gz

Resource identifier: ERR14888682

Data format : FASTQ

Download URL: ftp.sra.ebi.ac.uk/vol1/fastq/ERR148/082/ERR14888682/ERR14888682_2.fastq.gz

### Resource 5

Download URL: ftp.sra.ebi.ac.uk/vol1/fastq/ERR148/083/ERR14888683/ERR14888683_1.fastq.gz

Resource identifier: ERR14888683

Data format : FASTQ

Download URL: ftp.sra.ebi.ac.uk/vol1/fastq/ERR148/083/ERR14888683/ERR14888683_2.fastq.gz

### Resource 6

Download URL: ftp.sra.ebi.ac.uk/vol1/fastq/ERR148/084/ERR14888684/ERR14888684_1.fastq.gz

Resource identifier: ERR14888684

Data format : FASTQ

Download URL: ftp.sra.ebi.ac.uk/vol1/fastq/ERR148/084/ERR14888684/ERR14888684_2.fastq.gz

### Resource 7

Download URL: ftp.sra.ebi.ac.uk/vol1/fastq/ERR148/085/ERR14888685/ERR14888685_1.fastq.gz

Resource identifier: ERR14888685

Data format : FASTQ

Download URL: ftp.sra.ebi.ac.uk/vol1/fastq/ERR148/085/ERR14888685/ERR14888685_2.fastq.gz

### Resource 8

Download URL: ftp.sra.ebi.ac.uk/vol1/fastq/ERR148/067/ERR14888767/ERR14888767_1.fastq.gz

Resource identifier: ERR14888767

Data format : FASTQ

Download URL: ftp.sra.ebi.ac.uk/vol1/fastq/ERR148/067/ERR14888767/ERR14888767_2.fastq.gz

### Resource 9

Download URL: ftp.sra.ebi.ac.uk/vol1/fastq/ERR148/068/ERR14888768/ERR14888768_1.fastq.gz

Resource identifier: ERR14888768

Data format : FASTQ

Download URL: ftp.sra.ebi.ac.uk/vol1/fastq/ERR148/068/ERR14888768/ERR14888768_2.fastq.gz

### Resource 10

Download URL: ftp.sra.ebi.ac.uk/vol1/fastq/ERR148/069/ERR14888769/ERR14888769_1.fastq.gz

Resource identifier: ERR14888769

Data format : FASTQ

Download URL: ftp.sra.ebi.ac.uk/vol1/fastq/ERR148/069/ERR14888769/ERR14888769_2.fastq.gz

### Resource 11

Download URL: ftp.sra.ebi.ac.uk/vol1/fastq/ERR148/070/ERR14888770/ERR14888770_1.fastq.gz

Resource identifier: ERR14888770

Data format : FASTQ

Download URL: ftp.sra.ebi.ac.uk/vol1/fastq/ERR148/070/ERR14888770/ERR14888770_2.fastq.gz

### Resource 12

Download URL: ftp.sra.ebi.ac.uk/vol1/fastq/ERR148/071/ERR14888771/ERR14888771_1.fastq.gz

Resource identifier: ERR14888771

Data format : FASTQ

Download URL: ftp.sra.ebi.ac.uk/vol1/fastq/ERR148/071/ERR14888771/ERR14888771_2.fastq.gz

### Resource 13

Download URL: ftp.sra.ebi.ac.uk/vol1/fastq/ERR148/072/ERR14888772/ERR14888772_1.fastq.gz

Resource identifier: ERR14888772

Data format : FASTQ

Download URL: ftp.sra.ebi.ac.uk/vol1/fastq/ERR148/072/ERR14888772/ERR14888772_2.fastq.gz

### Resource 14

Download URL: ftp.sra.ebi.ac.uk/vol1/fastq/ERR148/073/ERR14888773/ERR14888773_1.fastq.gz

Resource identifier: ERR14888773

Data format : FASTQ

Download URL: ftp.sra.ebi.ac.uk/vol1/fastq/ERR148/073/ERR14888773/ERR14888773_2.fastq.gz

### Resource 15

Download URL: ftp.sra.ebi.ac.uk/vol1/fastq/ERR148/074/ERR14888774/ERR14888774_1.fastq.gz

Resource identifier: ERR14888774

Data format : FASTQ

Download URL: ftp.sra.ebi.ac.uk/vol1/fastq/ERR148/074/ERR14888774/ERR14888774_2.fastq.gz

### Resource 16

Download URL: ftp.sra.ebi.ac.uk/vol1/fastq/ERR148/075/ERR14888775/ERR14888775_1.fastq.gz

Resource identifier: ERR14888775

Data format : FASTQ

Download URL: ftp.sra.ebi.ac.uk/vol1/fastq/ERR148/075/ERR14888775/ERR14888775_2.fastq.gz

### Resource 17

Download URL: ftp.sra.ebi.ac.uk/vol1/fastq/ERR148/076/ERR14888776/ERR14888776_1.fastq.gz

Resource identifier: ERR14888776

Data format : FASTQ

Download URL: ftp.sra.ebi.ac.uk/vol1/fastq/ERR148/076/ERR14888776/ERR14888776_2.fastq.gz

### Resource 18

Download URL: ftp.sra.ebi.ac.uk/vol1/fastq/ERR148/077/ERR14888777/ERR14888777_1.fastq.gz

Resource identifier: ERR14888777

Data format : FASTQ

Download URL: ftp.sra.ebi.ac.uk/vol1/fastq/ERR148/077/ERR14888777/ERR14888777_2.fastq.gz

### Resource 19

Download URL: ftp.sra.ebi.ac.uk/vol1/fastq/ERR148/078/ERR14888778/ERR14888778_1.fastq.gz

Resource identifier: ERR14888778

Data format : FASTQ

Download URL: ftp.sra.ebi.ac.uk/vol1/fastq/ERR148/078/ERR14888778/ERR14888778_2.fastq.gz

### Resource 20

Download URL: ftp.sra.ebi.ac.uk/vol1/fastq/ERR148/088/ERR14888788/ERR14888788_1.fastq.gz

Resource identifier: ERR14888788

Data format : FASTQ

Download URL: ftp.sra.ebi.ac.uk/vol1/fastq/ERR148/088/ERR14888788/ERR14888788_2.fastq.gz

### Resource 21

Download URL: ftp.sra.ebi.ac.uk/vol1/fastq/ERR148/089/ERR14888789/ERR14888789_1.fastq.gz

Resource identifier: ERR14888789

Data format : FASTQ

Download URL: ftp.sra.ebi.ac.uk/vol1/fastq/ERR148/089/ERR14888789/ERR14888789_2.fastq.gz

### Resource 22

Download URL: ftp.sra.ebi.ac.uk/vol1/fastq/ERR148/090/ERR14888790/ERR14888790_1.fastq.gz

Resource identifier: ERR14888790

Data format : FASTQ

Download URL: ftp.sra.ebi.ac.uk/vol1/fastq/ERR148/090/ERR14888790/ERR14888790_2.fastq.gz

### Resource 23

Download URL: ftp.sra.ebi.ac.uk/vol1/fastq/ERR148/091/ERR14888791/ERR14888791_1.fastq.gz

Resource identifier: ERR14888791

Data format : FASTQ

Download URL: ftp.sra.ebi.ac.uk/vol1/fastq/ERR148/091/ERR14888791/ERR14888791_2.fastq.gz

### Resource 24

Download URL: ftp.sra.ebi.ac.uk/vol1/fastq/ERR148/092/ERR14888792/ERR14888792_1.fastq.gz

Resource identifier: ERR14888792

Data format : FASTQ

Download URL: ftp.sra.ebi.ac.uk/vol1/fastq/ERR148/092/ERR14888792/ERR14888792_2.fastq.gz

### Resource 25

Download URL: ftp.sra.ebi.ac.uk/vol1/fastq/ERR148/093/ERR14888793/ERR14888793_1.fastq.gz

Resource identifier: ERR14888793

Data format : FASTQ

Download URL: ftp.sra.ebi.ac.uk/vol1/fastq/ERR148/093/ERR14888793/ERR14888793_2.fastq.gz

### Resource 26

Download URL: ftp.sra.ebi.ac.uk/vol1/fastq/ERR148/094/ERR14888794/ERR14888794_1.fastq.gz

Resource identifier: ERR14888794

Data format : FASTQ

Download URL: ftp.sra.ebi.ac.uk/vol1/fastq/ERR148/094/ERR14888794/ERR14888794_2.fastq.gz

### Resource 27

Download URL: ftp.sra.ebi.ac.uk/vol1/fastq/ERR148/095/ERR14888795/ERR14888795_1.fastq.gz

Resource identifier: ERR14888795

Data format : FASTQ

Download URL: ftp.sra.ebi.ac.uk/vol1/fastq/ERR148/095/ERR14888795/ERR14888795_2.fastq.gz

### Resource 28

Download URL: ftp.sra.ebi.ac.uk/vol1/fastq/ERR148/059/ERR14888759/ERR14888759_1.fastq.gz

Resource identifier: ERR14888759

Data format : FASTQ

Download URL: ftp.sra.ebi.ac.uk/vol1/fastq/ERR148/059/ERR14888759/ERR14888759_2.fastq.gz

### Resource 29

Download URL: ftp.sra.ebi.ac.uk/vol1/fastq/ERR148/060/ERR14888760/ERR14888760_1.fastq.gz

Resource identifier: ERR14888760

Data format : FASTQ

Download URL: ftp.sra.ebi.ac.uk/vol1/fastq/ERR148/060/ERR14888760/ERR14888760_2.fastq.gz

### Resource 30

Download URL: ftp.sra.ebi.ac.uk/vol1/fastq/ERR148/061/ERR14888761/ERR14888761_1.fastq.gz

Resource identifier: ERR14888761

Data format : FASTQ

Download URL: ftp.sra.ebi.ac.uk/vol1/fastq/ERR148/061/ERR14888761/ERR14888761_2.fastq.gz

### Resource 31

Download URL: ftp.sra.ebi.ac.uk/vol1/fastq/ERR148/062/ERR14888762/ERR14888762_1.fastq.gz

Resource identifier: ERR14888762

Data format : FASTQ

Download URL: ftp.sra.ebi.ac.uk/vol1/fastq/ERR148/062/ERR14888762/ERR14888762_2.fastq.gz

### Resource 32

Download URL: ftp.sra.ebi.ac.uk/vol1/fastq/ERR148/063/ERR14888763/ERR14888763_1.fastq.gz

Resource identifier: ERR14888763

Data format : FASTQ

Download URL: ftp.sra.ebi.ac.uk/vol1/fastq/ERR148/063/ERR14888763/ERR14888763_2.fastq.gz

### Resource 33

Download URL: ftp.sra.ebi.ac.uk/vol1/fastq/ERR148/064/ERR14888764/ERR14888764_1.fastq.gz

Resource identifier: ERR14888764

Data format : FASTQ

Download URL: ftp.sra.ebi.ac.uk/vol1/fastq/ERR148/064/ERR14888764/ERR14888764_2.fastq.gz

### Resource 34

Download URL: ftp.sra.ebi.ac.uk/vol1/fastq/ERR148/065/ERR14888765/ERR14888765_1.fastq.gz

Resource identifier: ERR14888765

Data format : FASTQ

Download URL: ftp.sra.ebi.ac.uk/vol1/fastq/ERR148/065/ERR14888765/ERR14888765_2.fastq.gz

### Resource 35

Download URL: ftp.sra.ebi.ac.uk/vol1/fastq/ERR148/066/ERR14888766/ERR14888766_1.fastq.gz

Resource identifier: ERR14888766

Data format : FASTQ

Download URL: ftp.sra.ebi.ac.uk/vol1/fastq/ERR148/066/ERR14888766/ERR14888766_2.fastq.gz

### Resource 36

Download URL: ftp.sra.ebi.ac.uk/vol1/fastq/ERR148/096/ERR14888796/ERR14888796_1.fastq.gz

Resource identifier: ERR14888796

Data format : FASTQ

Download URL: ftp.sra.ebi.ac.uk/vol1/fastq/ERR148/096/ERR14888796/ERR14888796_2.fastq.gz

### Resource 37

Download URL: ftp.sra.ebi.ac.uk/vol1/fastq/ERR148/097/ERR14888797/ERR14888797_1.fastq.gz

Resource identifier: ERR14888797

Data format : FASTQ

Download URL: ftp.sra.ebi.ac.uk/vol1/fastq/ERR148/097/ERR14888797/ERR14888797_2.fastq.gz

### Resource 38

Download URL: ftp.sra.ebi.ac.uk/vol1/fastq/ERR148/098/ERR14888798/ERR14888798_1.fastq.gz

Resource identifier: ERR14888798

Data format : FASTQ

Download URL: ftp.sra.ebi.ac.uk/vol1/fastq/ERR148/098/ERR14888798/ERR14888798_2.fastq.gz

### Resource 39

Download URL: ftp.sra.ebi.ac.uk/vol1/fastq/ERR148/099/ERR14888799/ERR14888799_1.fastq.gz

Resource identifier: ERR14888799

Data format : FASTQ

Download URL: ftp.sra.ebi.ac.uk/vol1/fastq/ERR148/099/ERR14888799/ERR14888799_2.fastq.gz

### Resource 40

Download URL: ftp.sra.ebi.ac.uk/vol1/fastq/ERR148/000/ERR14888800/ERR14888800_1.fastq.gz

Resource identifier: ERR14888800

Data format : FASTQ

Download URL: ftp.sra.ebi.ac.uk/vol1/fastq/ERR148/000/ERR14888800/ERR14888800_2.fastq.gz

### Resource 41

Download URL: ftp.sra.ebi.ac.uk/vol1/fastq/ERR148/001/ERR14888801/ERR14888801_1.fastq.gz

Resource identifier: ERR14888801

Data format : FASTQ

Download URL: ftp.sra.ebi.ac.uk/vol1/fastq/ERR148/001/ERR14888801/ERR14888801_2.fastq.gz

### Resource 42

Download URL: ftp.sra.ebi.ac.uk/vol1/fastq/ERR148/002/ERR14888802/ERR14888802_1.fastq.gz

Resource identifier: ERR14888802

Data format : FASTQ

Download URL: ftp.sra.ebi.ac.uk/vol1/fastq/ERR148/002/ERR14888802/ERR14888802_2.fastq.gz

### Resource 43

Download URL: ftp.sra.ebi.ac.uk/vol1/fastq/ERR148/003/ERR14888803/ERR14888803_1.fastq.gz

Resource identifier: ERR14888803

Data format : FASTQ

Download URL: ftp.sra.ebi.ac.uk/vol1/fastq/ERR148/003/ERR14888803/ERR14888803_2.fastq.gz

### Resource 44

Download URL: ftp.sra.ebi.ac.uk/vol1/fastq/ERR148/004/ERR14888804/ERR14888804_1.fastq.gz

Resource identifier: ERR14888804

Data format : FASTQ

Download URL: ftp.sra.ebi.ac.uk/vol1/fastq/ERR148/004/ERR14888804/ERR14888804_2.fastq.gz

### Resource 45

Download URL: ftp.sra.ebi.ac.uk/vol1/fastq/ERR148/005/ERR14888805/ERR14888805_1.fastq.gz

Resource identifier: ERR14888805

Data format : FASTQ

Download URL: ftp.sra.ebi.ac.uk/vol1/fastq/ERR148/005/ERR14888805/ERR14888805_2.fastq.gz

### Resource 46

Download URL: ftp.sra.ebi.ac.uk/vol1/fastq/ERR148/006/ERR14888806/ERR14888806_1.fastq.gz

Resource identifier: ERR14888806

Data format : FASTQ

Download URL: ftp.sra.ebi.ac.uk/vol1/fastq/ERR148/006/ERR14888806/ERR14888806_2.fastq.gz

### Resource 47

Download URL: ftp.sra.ebi.ac.uk/vol1/fastq/ERR148/007/ERR14888807/ERR14888807_1.fastq.gz

Resource identifier: ERR14888807

Data format : FASTQ

Download URL: ftp.sra.ebi.ac.uk/vol1/fastq/ERR148/007/ERR14888807/ERR14888807_2.fastq.gz

### Resource 48

Download URL: ftp.sra.ebi.ac.uk/vol1/fastq/ERR148/008/ERR14888808/ERR14888808_1.fastq.gz

Resource identifier: ERR14888808

Data format : FASTQ

Download URL: ftp.sra.ebi.ac.uk/vol1/fastq/ERR148/008/ERR14888808/ERR14888808_2.fastq.gz

### Resource 49

Download URL: ftp.sra.ebi.ac.uk/vol1/fastq/ERR148/009/ERR14888809/ERR14888809_1.fastq.gz

Resource identifier: ERR14888809

Data format : FASTQ

Download URL: ftp.sra.ebi.ac.uk/vol1/fastq/ERR148/009/ERR14888809/ERR14888809_2.fastq.gz

### Resource 50

Download URL: ftp.sra.ebi.ac.uk/vol1/fastq/ERR148/010/ERR14888810/ERR14888810_1.fastq.gz

Resource identifier: ERR14888810

Data format : FASTQ

Download URL: ftp.sra.ebi.ac.uk/vol1/fastq/ERR148/010/ERR14888810/ERR14888810_2.fastq.gz

### Resource 51

Download URL: ftp.sra.ebi.ac.uk/vol1/fastq/ERR148/011/ERR14888811/ERR14888811_1.fastq.gz

Resource identifier: ERR14888811

Data format : FASTQ

Download URL: ftp.sra.ebi.ac.uk/vol1/fastq/ERR148/011/ERR14888811/ERR14888811_2.fastq.gz

### Resource 52

Download URL: ftp.sra.ebi.ac.uk/vol1/fastq/ERR148/097/ERR14888897/ERR14888897_1.fastq.gz

Resource identifier: ERR14888897

Data format : FASTQ

Download URL: ftp.sra.ebi.ac.uk/vol1/fastq/ERR148/097/ERR14888897/ERR14888897_2.fastq.gz

### Resource 53

Download URL: ftp.sra.ebi.ac.uk/vol1/fastq/ERR148/098/ERR14888898/ERR14888898_1.fastq.gz

Resource identifier: ERR14888898

Data format : FASTQ

Download URL: ftp.sra.ebi.ac.uk/vol1/fastq/ERR148/098/ERR14888898/ERR14888898_2.fastq.gz

### Resource 54

Download URL: ftp.sra.ebi.ac.uk/vol1/fastq/ERR148/099/ERR14888899/ERR14888899_1.fastq.gz

Resource identifier: ERR14888899

Data format : FASTQ

Download URL: ftp.sra.ebi.ac.uk/vol1/fastq/ERR148/099/ERR14888899/ERR14888899_2.fastq.gz

### Resource 55

Download URL: ftp.sra.ebi.ac.uk/vol1/fastq/ERR148/000/ERR14888900/ERR14888900_1.fastq.gz

Resource identifier: ERR14888900

Data format : FASTQ

Download URL: ftp.sra.ebi.ac.uk/vol1/fastq/ERR148/000/ERR14888900/ERR14888900_2.fastq.gz

### Resource 56

Download URL: ftp.sra.ebi.ac.uk/vol1/fastq/ERR148/001/ERR14888901/ERR14888901_1.fastq.gz

Resource identifier: ERR14888901

Data format : FASTQ

Download URL: ftp.sra.ebi.ac.uk/vol1/fastq/ERR148/001/ERR14888901/ERR14888901_2.fastq.gz

### Resource 57

Download URL: ftp.sra.ebi.ac.uk/vol1/fastq/ERR148/002/ERR14888902/ERR14888902_1.fastq.gz

Resource identifier: ERR14888902

Data format : FASTQ

Download URL: ftp.sra.ebi.ac.uk/vol1/fastq/ERR148/002/ERR14888902/ERR14888902_2.fastq.gz

### Resource 58

Download URL: ftp.sra.ebi.ac.uk/vol1/fastq/ERR148/003/ERR14888903/ERR14888903_1.fastq.gz

Resource identifier: ERR14888903

Data format : FASTQ

Download URL: ftp.sra.ebi.ac.uk/vol1/fastq/ERR148/003/ERR14888903/ERR14888903_2.fastq.gz

### Resource 59

Download URL: ftp.sra.ebi.ac.uk/vol1/fastq/ERR148/004/ERR14888904/ERR14888904_1.fastq.gz

Resource identifier: ERR14888904

Data format : FASTQ

Download URL: ftp.sra.ebi.ac.uk/vol1/fastq/ERR148/004/ERR14888904/ERR14888904_2.fastq.gz

### Resource 60

Download URL: ftp.sra.ebi.ac.uk/vol1/fastq/ERR148/005/ERR14888905/ERR14888905_1.fastq.gz

Resource identifier: ERR14888905

Data format : FASTQ

Download URL: ftp.sra.ebi.ac.uk/vol1/fastq/ERR148/005/ERR14888905/ERR14888905_2.fastq.gz

### Resource 61

Download URL: ftp.sra.ebi.ac.uk/vol1/fastq/ERR148/006/ERR14888906/ERR14888906_1.fastq.gz

Resource identifier: ERR14888906

Data format : FASTQ

Download URL: ftp.sra.ebi.ac.uk/vol1/fastq/ERR148/006/ERR14888906/ERR14888906_2.fastq.gz

### Resource 62

Download URL: ftp.sra.ebi.ac.uk/vol1/fastq/ERR148/007/ERR14888907/ERR14888907_1.fastq.gz

Resource identifier: ERR14888907

Data format : FASTQ

Download URL: ftp.sra.ebi.ac.uk/vol1/fastq/ERR148/007/ERR14888907/ERR14888907_2.fastq.gz

### Resource 63

Download URL: ftp.sra.ebi.ac.uk/vol1/fastq/ERR148/008/ERR14888908/ERR14888908_1.fastq.gz

Resource identifier: ERR14888908

Data format : FASTQ

Download URL: ftp.sra.ebi.ac.uk/vol1/fastq/ERR148/008/ERR14888908/ERR14888908_2.fastq.gz

### Resource 64

Download URL: ftp.sra.ebi.ac.uk/vol1/fastq/ERR148/000/ERR14888600/ERR14888600_1.fastq.gz

Resource identifier: ERR14888600

Data format : FASTQ

Download URL: ftp.sra.ebi.ac.uk/vol1/fastq/ERR148/000/ERR14888600/ERR14888600_2.fastq.gz

### Resource 65

Download URL: ftp.sra.ebi.ac.uk/vol1/fastq/ERR148/001/ERR14888601/ERR14888601_1.fastq.gz

Resource identifier: ERR14888601

Data format : FASTQ

Download URL: ftp.sra.ebi.ac.uk/vol1/fastq/ERR148/001/ERR14888601/ERR14888601_2.fastq.gz

### Resource 66

Download URL: ftp.sra.ebi.ac.uk/vol1/fastq/ERR148/079/ERR14888779/ERR14888779_1.fastq.gz

Resource identifier: ERR14888779

Data format : FASTQ

Download URL: ftp.sra.ebi.ac.uk/vol1/fastq/ERR148/079/ERR14888779/ERR14888779_2.fastq.gz

### Resource 67

Download URL: ftp.sra.ebi.ac.uk/vol1/fastq/ERR148/080/ERR14888780/ERR14888780_1.fastq.gz

Resource identifier: ERR14888780

Data format : FASTQ

Download URL: ftp.sra.ebi.ac.uk/vol1/fastq/ERR148/080/ERR14888780/ERR14888780_2.fastq.gz

### Resource 68

Download URL: ftp.sra.ebi.ac.uk/vol1/fastq/ERR148/081/ERR14888781/ERR14888781_1.fastq.gz

Resource identifier: ERR14888781

Data format : FASTQ

Download URL: ftp.sra.ebi.ac.uk/vol1/fastq/ERR148/081/ERR14888781/ERR14888781_2.fastq.gz

### Resource 69

Download URL: ftp.sra.ebi.ac.uk/vol1/fastq/ERR148/082/ERR14888782/ERR14888782_1.fastq.gz

Resource identifier: ERR14888782

Data format : FASTQ

Download URL: ftp.sra.ebi.ac.uk/vol1/fastq/ERR148/082/ERR14888782/ERR14888782_2.fastq.gz

### Resource 70

Download URL: ftp.sra.ebi.ac.uk/vol1/fastq/ERR148/083/ERR14888783/ERR14888783_1.fastq.gz

Resource identifier: ERR14888783

Data format : FASTQ

Download URL: ftp.sra.ebi.ac.uk/vol1/fastq/ERR148/083/ERR14888783/ERR14888783_2.fastq.gz

### Resource 71

Download URL: ftp.sra.ebi.ac.uk/vol1/fastq/ERR148/084/ERR14888784/ERR14888784_1.fastq.gz

Resource identifier: ERR14888784

Data format : FASTQ

Download URL: ftp.sra.ebi.ac.uk/vol1/fastq/ERR148/084/ERR14888784/ERR14888784_2.fastq.gz

### Resource 72

Download URL: ftp.sra.ebi.ac.uk/vol1/fastq/ERR148/085/ERR14888785/ERR14888785_1.fastq.gz

Resource identifier: ERR14888785

Data format : FASTQ

Download URL: ftp.sra.ebi.ac.uk/vol1/fastq/ERR148/085/ERR14888785/ERR14888785_2.fastq.gz

### Resource 73

Download URL: ftp.sra.ebi.ac.uk/vol1/fastq/ERR148/086/ERR14888786/ERR14888786_1.fastq.gz

Resource identifier: ERR14888786

Data format : FASTQ

Download URL: ftp.sra.ebi.ac.uk/vol1/fastq/ERR148/086/ERR14888786/ERR14888786_2.fastq.gz

### Resource 74

Download URL: ftp.sra.ebi.ac.uk/vol1/fastq/ERR148/070/ERR14888970/ERR14888970_1.fastq.gz

Resource identifier: ERR14888970

Data format : FASTQ

Download URL: ftp.sra.ebi.ac.uk/vol1/fastq/ERR148/070/ERR14888970/ERR14888970_2.fastq.gz

### Resource 75

Download URL: ftp.sra.ebi.ac.uk/vol1/fastq/ERR148/072/ERR14888972/ERR14888972_1.fastq.gz

Resource identifier: ERR14888972

Data format : FASTQ

Download URL: ftp.sra.ebi.ac.uk/vol1/fastq/ERR148/072/ERR14888972/ERR14888972_2.fastq.gz

### Resource 76

Download URL: ftp.sra.ebi.ac.uk/vol1/fastq/ERR148/073/ERR14888973/ERR14888973_1.fastq.gz

Resource identifier: ERR14888973

Data format : FASTQ

Download URL: ftp.sra.ebi.ac.uk/vol1/fastq/ERR148/073/ERR14888973/ERR14888973_2.fastq.gz

### Resource 77

Download URL: ftp.sra.ebi.ac.uk/vol1/fastq/ERR148/074/ERR14888974/ERR14888974_1.fastq.gz

Resource identifier: ERR14888974

Data format : FASTQ

Download URL: ftp.sra.ebi.ac.uk/vol1/fastq/ERR148/074/ERR14888974/ERR14888974_2.fastq.gz

### Resource 78

Download URL: ftp.sra.ebi.ac.uk/vol1/fastq/ERR148/075/ERR14888975/ERR14888975_1.fastq.gz

Resource identifier: ERR14888975

Data format : FASTQ

Download URL: ftp.sra.ebi.ac.uk/vol1/fastq/ERR148/075/ERR14888975/ERR14888975_2.fastq.gz

### Resource 79

Download URL: ftp.sra.ebi.ac.uk/vol1/fastq/ERR148/076/ERR14888976/ERR14888976_1.fastq.gz

Resource identifier: ERR14888976

Data format : FASTQ

Download URL: ftp.sra.ebi.ac.uk/vol1/fastq/ERR148/076/ERR14888976/ERR14888976_2.fastq.gz

### Resource 80

Download URL: ftp.sra.ebi.ac.uk/vol1/fastq/ERR148/077/ERR14888977/ERR14888977_1.fastq.gz

Resource identifier: ERR14888977

Data format : FASTQ

Download URL: ftp.sra.ebi.ac.uk/vol1/fastq/ERR148/077/ERR14888977/ERR14888977_2.fastq.gz

### Resource 81

Download URL: ftp.sra.ebi.ac.uk/vol1/fastq/ERR148/078/ERR14888978/ERR14888978_1.fastq.gz

Resource identifier: ERR14888978

Data format : FASTQ

Download URL: ftp.sra.ebi.ac.uk/vol1/fastq/ERR148/078/ERR14888978/ERR14888978_2.fastq.gz

### Resource 82

Download URL: ftp.sra.ebi.ac.uk/vol1/fastq/ERR148/079/ERR14888979/ERR14888979_1.fastq.gz

Resource identifier: ERR14888979

Data format : FASTQ

Download URL: ftp.sra.ebi.ac.uk/vol1/fastq/ERR148/079/ERR14888979/ERR14888979_2.fastq.gz

### Resource 83

Download URL: ftp.sra.ebi.ac.uk/vol1/fastq/ERR148/080/ERR14888980/ERR14888980_1.fastq.gz

Resource identifier: ERR14888980

Data format : FASTQ

Download URL: ftp.sra.ebi.ac.uk/vol1/fastq/ERR148/080/ERR14888980/ERR14888980_2.fastq.gz

### Resource 84

Download URL: ftp.sra.ebi.ac.uk/vol1/fastq/ERR148/081/ERR14888981/ERR14888981_1.fastq.gz

Resource identifier: ERR14888981

Data format : FASTQ

Download URL: ftp.sra.ebi.ac.uk/vol1/fastq/ERR148/081/ERR14888981/ERR14888981_2.fastq.gz

### Resource 85

Download URL: ftp.sra.ebi.ac.uk/vol1/fastq/ERR148/082/ERR14888982/ERR14888982_1.fastq.gz

Resource identifier: ERR14888982

Data format : FASTQ

Download URL: ftp.sra.ebi.ac.uk/vol1/fastq/ERR148/082/ERR14888982/ERR14888982_2.fastq.gz

### Resource 86

Download URL: ftp.sra.ebi.ac.uk/vol1/fastq/ERR148/058/ERR14888958/ERR14888958_1.fastq.gz

Resource identifier: ERR14888958

Data format : FASTQ

Download URL: ftp.sra.ebi.ac.uk/vol1/fastq/ERR148/058/ERR14888958/ERR14888958_2.fastq.gz

### Resource 87

Download URL: ftp.sra.ebi.ac.uk/vol1/fastq/ERR148/059/ERR14888959/ERR14888959_1.fastq.gz

Resource identifier: ERR14888959

Data format : FASTQ

Download URL: ftp.sra.ebi.ac.uk/vol1/fastq/ERR148/059/ERR14888959/ERR14888959_2.fastq.gz

### Resource 88

Download URL: ftp.sra.ebi.ac.uk/vol1/fastq/ERR148/060/ERR14888960/ERR14888960_1.fastq.gz

Resource identifier: ERR14888960

Data format : FASTQ

Download URL: ftp.sra.ebi.ac.uk/vol1/fastq/ERR148/060/ERR14888960/ERR14888960_2.fastq.gz

### Resource 89

Download URL: ftp.sra.ebi.ac.uk/vol1/fastq/ERR148/061/ERR14888961/ERR14888961_1.fastq.gz

Resource identifier: ERR14888961

Data format : FASTQ

Download URL: ftp.sra.ebi.ac.uk/vol1/fastq/ERR148/061/ERR14888961/ERR14888961_2.fastq.gz

### Resource 90

Download URL: ftp.sra.ebi.ac.uk/vol1/fastq/ERR148/062/ERR14888962/ERR14888962_1.fastq.gz

Resource identifier: ERR14888962

Data format : FASTQ

Download URL: ftp.sra.ebi.ac.uk/vol1/fastq/ERR148/062/ERR14888962/ERR14888962_2.fastq.gz

### Resource 91

Download URL: ftp.sra.ebi.ac.uk/vol1/fastq/ERR148/063/ERR14888963/ERR14888963_1.fastq.gz

Resource identifier: ERR14888963

Data format : FASTQ

Download URL: ftp.sra.ebi.ac.uk/vol1/fastq/ERR148/063/ERR14888963/ERR14888963_2.fastq.gz

### Resource 92

Download URL: ftp.sra.ebi.ac.uk/vol1/fastq/ERR148/064/ERR14888964/ERR14888964_1.fastq.gz

Resource identifier: ERR14888964

Data format : FASTQ

Download URL: ftp.sra.ebi.ac.uk/vol1/fastq/ERR148/064/ERR14888964/ERR14888964_2.fastq.gz

### Resource 93

Download URL: ftp.sra.ebi.ac.uk/vol1/fastq/ERR148/065/ERR14888965/ERR14888965_1.fastq.gz

Resource identifier: ERR14888965

Data format : FASTQ

Download URL: ftp.sra.ebi.ac.uk/vol1/fastq/ERR148/065/ERR14888965/ERR14888965_2.fastq.gz

### Resource 94

Download URL: ftp.sra.ebi.ac.uk/vol1/fastq/ERR148/066/ERR14888966/ERR14888966_1.fastq.gz

Resource identifier: ERR14888966

Data format : FASTQ

Download URL: ftp.sra.ebi.ac.uk/vol1/fastq/ERR148/066/ERR14888966/ERR14888966_2.fastq.gz

### Resource 95

Download URL: ftp.sra.ebi.ac.uk/vol1/fastq/ERR148/067/ERR14888967/ERR14888967_1.fastq.gz

Resource identifier: ERR14888967

Data format : FASTQ

Download URL: ftp.sra.ebi.ac.uk/vol1/fastq/ERR148/067/ERR14888967/ERR14888967_.fastq.gz

### Resource 96

Download URL: ftp.sra.ebi.ac.uk/vol1/fastq/ERR148/068/ERR14888968/ERR14888968_1.fastq.gz

Resource identifier: ERR14888968

Data format : FASTQ

Download URL: ftp.sra.ebi.ac.uk/vol1/fastq/ERR148/068/ERR14888968/ERR14888968_2.fastq.gz

### Resource 97

Download URL: ftp.sra.ebi.ac.uk/vol1/fastq/ERR148/069/ERR14888969/ERR14888969_1.fastq.gz

Resource identifier: ERR14888969

Data format : FASTQ

Download URL: ftp.sra.ebi.ac.uk/vol1/fastq/ERR148/069/ERR14888969/ERR14888969_2.fastq.gz

### Resource 98

Download URL: ftp.sra.ebi.ac.uk/vol1/fastq/ERR148/014/ERR14888714/ERR14888714_1.fastq.gz

Resource identifier: ERR14888714

Data format : FASTQ

Download URL: ftp.sra.ebi.ac.uk/vol1/fastq/ERR148/014/ERR14888714/ERR14888714_2.fastq.gz

### Resource 99

Download URL: ftp.sra.ebi.ac.uk/vol1/fastq/ERR148/015/ERR14888715/ERR14888715_1.fastq.gz

Resource identifier: ERR14888715

Data format : FASTQ

Download URL: ftp.sra.ebi.ac.uk/vol1/fastq/ERR148/015/ERR14888715/ERR14888715_2.fastq.gz

### Resource 100

Download URL: ftp.sra.ebi.ac.uk/vol1/fastq/ERR148/049/ERR14888849/ERR14888849_1.fastq.gz

Resource identifier: ERR14888849

Data format : FASTQ

Download URL: ftp.sra.ebi.ac.uk/vol1/fastq/ERR148/049/ERR14888849/ERR14888849_2.fastq.gz

### Resource 101

Download URL: ftp.sra.ebi.ac.uk/vol1/fastq/ERR148/050/ERR14888850/ERR14888850_1.fastq.gz

Resource identifier: ERR14888850

Data format : FASTQ

Download URL: ftp.sra.ebi.ac.uk/vol1/fastq/ERR148/050/ERR14888850/ERR14888850_2.fastq.gz

### Resource 102

Download URL: ftp.sra.ebi.ac.uk/vol1/fastq/ERR148/051/ERR14888851/ERR14888851_1.fastq.gz

Resource identifier: ERR14888851

Data format : FASTQ

Download URL: ftp.sra.ebi.ac.uk/vol1/fastq/ERR148/051/ERR14888851/ERR14888851_2.fastq.gz

### Resource 103

Download URL: ftp.sra.ebi.ac.uk/vol1/fastq/ERR148/052/ERR14888852/ERR14888852_1.fastq.gz

Resource identifier: ERR14888852

Data format : FASTQ

Download URL: ftp.sra.ebi.ac.uk/vol1/fastq/ERR148/052/ERR14888852/ERR14888852_.fastq.gz

### Resource 104

Download URL: ftp.sra.ebi.ac.uk/vol1/fastq/ERR148/053/ERR14888853/ERR14888853_1.fastq.gz

Resource identifier: ERR14888853

Data format : FASTQ

Download URL: ftp.sra.ebi.ac.uk/vol1/fastq/ERR148/053/ERR14888853/ERR14888853_2.fastq.gz

### Resource 105

Download URL: ftp.sra.ebi.ac.uk/vol1/fastq/ERR148/054/ERR14888854/ERR14888854_1.fastq.gz

Resource identifier: ERR14888854

Data format : FASTQ

Download URL: ftp.sra.ebi.ac.uk/vol1/fastq/ERR148/054/ERR14888854/ERR14888854_.fastq.gz

### Resource 106

Download URL: ftp.sra.ebi.ac.uk/vol1/fastq/ERR148/055/ERR14888855/ERR14888855_1.fastq.gz

Resource identifier: ERR14888855

Data format : FASTQ

Download URL: ftp.sra.ebi.ac.uk/vol1/fastq/ERR148/055/ERR14888855/ERR14888855_2.fastq.gz

### Resource 107

Download URL: ftp.sra.ebi.ac.uk/vol1/fastq/ERR148/056/ERR14888856/ERR14888856_1.fastq.gz

Resource identifier: ERR14888856

Data format : FASTQ

Download URL: ftp.sra.ebi.ac.uk/vol1/fastq/ERR148/056/ERR14888856/ERR14888856_2.fastq.gz

### Resource 108

Download URL: ftp.sra.ebi.ac.uk/vol1/fastq/ERR148/079/ERR14888679/ERR14888679_1.fastq.gz

Resource identifier: ERR14888679

Data format : FASTQ

Download URL: ftp.sra.ebi.ac.uk/vol1/fastq/ERR148/079/ERR14888679/ERR14888679_2.fastq.gz

## Usage Rights

CC BY 4.0

Usage Rights

## Supplementary Material

A4764E50-8859-5E8C-9D12-A031D6FEB509 10.3897/BDJ.14.e178484.suppl1 Supplementary material 1 ENA accession numbers Data type metadata Brief description ENA accession numbers (sample, experiment, run, project and umbrella project) for the EMO BON shotgun metagenomics data release from seawater and sediment samples (batch_2, second release). File: oo_1460327.csv https://binary.pensoft.net/file/1460327 Christina Pavloudi

## Figures and Tables

**Table 1.** Coordinates, locality, sampling type (seawater and/or sediment) and number of samples for the operational observatories sampling sites.

Observatory	Coordinates	Ocean/Sea	Region	Location	Marine Ecoregion of the World (MEOW)	Seawater sampling	Sediment sampling	Total Number of samples in this batch	Number of successfully sequenced samples
AAOT	45.31417N; 12.508333E	Mediterranean Sea - Eastern Basin	Adriatic Sea	Gulf of Venice	Adriatic Sea	Yes		8	8
BPNS	51.43333N; 2.808331E	North Atlantic Ocean	North Sea	Belgian part of the North Sea	North Sea	Yes	Yes	12	12
EMT21	42.20194N; -8.798500W	Atlantic Ocean	North Atlantic Ocean	Vigo Seamount	South European Atlantic Shelf	Yes		8	8
ESC68N	68.92589N; 17.125619E	Arctic Ocean	Norwegian Sea	Norwegian part of the Norwegian Sea	Northern Norway and Finnmark	Yes		8	2
IUIEilat	29.50000N; 34.916667E	Indian Ocean	Gulf of Eilat	Gulf of Eilat	Northern and Central Red Sea	Yes		8	8
NRMCB	40.80014N; 14.250000E	Mediterranean Sea - Western Basin	Tyrrhenian Sea	Naples Gulf	Western Mediterranean	Yes	Yes	8	12
OOB	42.489N; 3.143E	Mediterranean Sea - Western Basin	Gulf of Lion	Bay of Banyuls-sur-Mer	Western Mediterranean		Yes	4	2
OSD74	41.14653N; -8.666639W	Atlantic Ocean	North Atlantic Ocean	Porto Valley	South European Atlantic Shelf	Yes		8	8
PiEGetxo	43.33858N; -3.014639W	North Atlantic Ocean	Bay of Biscay	Abra de Bilbao	South European Atlantic Shelf	Yes		8	8
RFormosa	37.00564N; -7.969250W	Atlantic Ocean	North Atlantic Ocean	Ria Formosa	South European Atlantic Shelf	Yes	Yes	12	12
ROSKOGO	48.70833N; -3.866000W	North Atlantic Ocean	English Channel	French part of the English Channel	Celtic Seas		Yes	4	4
ROSKOGO	48.77167N; -3.968333W	North Atlantic Ocean	English Channel	French part of the English Channel	Celtic Seas	Yes		8	8
VB	43.68300N; 7.317000E	Mediterranean Sea - Western Basin	Villefranche Bay	Villefranche Bay - Point B	Western Mediterranean	Yes		8	8
MBAL4	50.25N; -4.217W	North Atlantic Ocean	English Channel	Western Channel	Celtic Seas	Yes		8	8
